# Complex Association between Alanine Aminotransferase Activity and Mortality in General Population: A Systematic Review and Meta-Analysis of Prospective Studies

**DOI:** 10.1371/journal.pone.0091410

**Published:** 2014-03-14

**Authors:** Zhengtao Liu, Huaijun Ning, Shuping Que, Linlin Wang, Xue Qin, Tao Peng

**Affiliations:** 1 Department of Hepatobiliary Surgery, First Affiliated Hospital of Guangxi Medical University, Nanning, China; 2 National Center for International Research of Biological Targeting Diagnosis and Therapy, Guangxi Medical University, Nanning, China; 3 Department of Pediatrics, Women and children's hospital of Guangxi, Nanning, China; 4 Department of Pediatrics, First Affiliated Hospital of Guangxi Medical University, Nanning, China; 5 Clinical Laboratory Center, First Affiliated Hospital of Guangxi Medical University, Nanning, China; 6 Guangxi Key Laboratory of Biological Targeting Diagnosis and Therapy Research, Guangxi Medical University, Nanning, China; University of North Carolina School of Medicine, United States of America

## Abstract

**Objective:**

Controversy exists in using alanine aminotransferase (ALT) activity for predicting long-term survival. Therefore, this research study investigated the association between ALT activity and mortality through a systematic review and meta-analysis of previous prospective studies.

**Methods:**

Electronic literature databases, including PubMed, Embase, and the Institute for Scientific Information (ISI), were searched for relevant prospective observational studies (published before Dec 30, 2013) on the association between baseline ALT activity and ensuing all-cause/disease-specific mortality. Information on nationality, sample size, participant characteristics, follow-up duration, comparison, outcome assessment, hazard ratios (HRs) and adjusted covariates was extracted. Pooled HRs and corresponding 95% confidence intervals (CIs) were separately calculated for categorical risk estimates (highest vs. lowest ALT categories) and continuous risk estimates (per 5 U/l of ALT increment) in subgroups separated by age (<70/≥70 years).

**Results:**

A total of twelve prospective cohort studies, totaling 206,678 participants and 16,249 deaths, were identified and analyzed. In the younger age group, the pooled HR for mortality related to liver-disease was about 1.24 (95% CI: 1.23–1.25) per 5 U/l of ALT increment. The dose-response HRs of all-cause mortality, cardiovascular (CV) disease-related mortality, and cancer-related mortality were 0.91 (0.88–0.94), 0.91 (0.85–0.96), 0.92 (0.86–0.98) respectively per 5 U/l of ALT elevation, with insignificant heterogeneity in the older population. There was an approximate decrease of 4‰ observed on HRs of all-cause, CV-related, and cancer-related mortality followed with one year's increment through meta-regression (all P<0.05).

**Conclusions:**

The ALT-mortality association was inconsistent and seems particularly susceptible to age after synthesizing the previous prospective studies. In terms of the age, ALT activity was more valuable in predicting mortality in the older population; extremely low ALT levels indicated a higher all-cause, CV-related, and cancer-related mortality. ALT activity may therefore be a useful biomarker when predicting the long-term survival of elderly patients.

## Introduction

Measurements of serum alanine aminotransferase (ALT) activity is an available and low-cost biochemical assay in annual health check-ups. Serum ALT activity is not only a biomarker for monitoring liver damage, but is also a predictor for evaluating overall health [1], [2]. In previous epidemiological prospective cohort surveys [3]–[5], elevated ALT activity was associated with all-cause/cause specific (including cardiovascular (CV) disease, cancer, and liver disease) mortality in long-term follow-up duration. The elevated ALT activity was potentially indicative of unrecognized liver disease that especially caused deaths in populations with a high prevalence of viral hepatitis [3]. On the other hand, elevated ALT activity was a potential predictor of CV disease that might add to a risk of death through the metabolic syndrome (MetS) [6], [7]. However, these associations have been inconsistent and controversial as some prospective studies have failed to observe a significant association between ALT activity and mortality [8]–[14].

Recently, lower ALT activity was linked to decreased long-term survival in older population [13], [15], [17]–[19]. The clinical significance of extra low ALT activity as a reliable biomarker for predicting increased mortality, especially in aging population (mean age≥70 years) [13], [15]–[18], has caused worldwide concern. The possible interpretation is that the lower ALT activity indicates aging and frailty [19]–[22] in overall health, decreased size and reduced blood flow of the liver [23], [24], which might increase susceptibility to a number of diseases.

The association between ALT activity and mortality is complex, inconsistent and controversial. One study that examined prior prospective studies indicated that increased age and ensuing frailty might be a possible confounder in the ALT-mortality association [25]. Age may play a crucial role in mediating the ALT-mortality association [26]; however, the pooled risks of mortality caused by ALT variation and the exact effects of age on this association have never been assessed. Therefore, a systematic review and meta-analysis of previous prospective cohort studies classified by mean age (<70/≥70 years) was performed to evaluate the pooled effect of ALT activity on the risk of all-cause/cause-specific mortality. In addition, the exact effect of age on this association was quantified through meta-regression. Furthermore, subgroup and sensitivity analysis was conducted to search for other potential confounders that might cause heterogeneity in synthetic analysis. To the best of our knowledge, this is the first systematic review and meta-analysis to evaluate the association between ALT activity and all-cause/disease- specific mortality.

## Materials and Methods

### PRISMA and flow diagram

The meta-analysis was conducted according to the guidelines of the Preferred Reporting Items for Systematic Reviews and Meta-Analyses (PRISMA) [27]. The supporting PRISMA checklist and flow diagram for this meta-analysis is available as supporting information; see Checklist S1 and Flow Diagram S1, respectively.

### Search strategy and study selection

An extensive relevant literature search was conducted in PubMed, Embase and the Institute for Scientific Information (ISI) database, without language restrictions, through help from a qualified librarian (last updated at Dec 30th, 2013). The search was restricted to studies conducted in human subjects. Medical subject headings “alanine aminotransferase”, “transaminase”, “liver enzyme”, “ALT”, and “mortality” were used for searching the relevant literatures. The following search terms were used in PubMed: (alanine aminotransferase [All Fields] OR alanine aminotransferase [MeSH] OR transaminase [MESH] OR transaminase [All Fields] AND mortality [MeSH]). Similar search terms were applied to Embase and ISI (see Table S1). In addition, a manual search of citations from relevant original studies and review articles was performed. The reporting of this systematic review and meta-analysis were performed in accordance with the Meta-analysis of Observational Studies in Epidemiology (MOOSE) guidelines [28].

Only reports fulfilling the following inclusion criteria were included in the systematic review and meta-analysis: 1) prospective cohort studies published with original data, 2) ALT levels and mean age range of study participants at baseline were reported, and all-cause (essential) and/or cause-specific (including CV, cancer, and liver disease; dispensable) related mortality was an end point associated with ALT activity, 3) the hazard ratio (HR) or relative risk (RR) of mortality in the population with the lowest ALT category was treated as reference data, 4) studies that contained the minimum information necessary to estimate the continuous or dichotomous HR or RR of all-cause/cause-specific mortality caused by ALT elevation compared in the same population were provided or obtained by calculation. If data were duplicated in more than one study, the most recent study was included in the analysis. Literature reviews, retrospective cohort studies, twins studies, case-control studies, case-cohort studies, and animal studies were excluded.

### Quality assessment

The quality of each study was independently assessed through two authors using the Newcastle-Ottawa Scale (NOS) [29].The NOS consists of three parameters of quality: selection, comparability, and outcome for cohort studies (Table S2). The NOS assigned a maximum of four stars for selection, two stars for comparability, and three stars for outcome. Therefore, nine stars reflected the highest quality. Studies with more than six stars were considered high quality. When discrepancies occurred, a joint revaluation of the original article was performed with a third author.

### Outcome measures and data extraction

Mortality outcomes consisted of all-cause mortality and the following cause-specific mortality were based on death certificates with underlying cause of death coded according to the International Classification of Diseases, Ninth Revision (ICD-9) [30]: CV mortality included the following subgroups according to ICD-9: coronary heart disease (ICD-9 410 to 414), congestive heart failure (ICD-9 428, 429), other cardiovascular disease (ICD-9 401 to 405, 440 to 443), stroke (ICD-9 430 to 438), cancer mortality (ICD-9 140 –239), and liver disease mortality (ICD-9 70.2–70.9, 275.0–275.1, and 570–573).

Two authors independently extracted information using a standardized reporting form with predefined criteria specifically for this review. Study characteristics recorded were extracted as follows: first author, publication year, country of origin, study characteristics (numbers of participants and cases, definition of normal ALT value and percentage of participants in normal range, gender category, mean age range of populations, measures of outcome, follow-up durations, comparison style, adjusted HR or RR of mortality estimated from multivariable analysis according to the comparison style referred before and adjusted covariates in detail). When effects estimated in the same population were reported in different follow-up durations, only data with the longest follow-up time were included.

### Statistical analysis

HR was selected as the effect to assess the association between ALT and all-cause/disease specific mortality. RR was treated as HR. Data analyses used the most multivariate-adjusted HR and 95% CI. If the publications reported separate HR for gender or age, the pooled HRs were separately pooled as independent data points in subsequent meta-analysis. Otherwise, the separate HRs were combined.

To distinguish the clinical significance of ALT activity in different age groups, all of the enrolled studies were divided into two separate groups by mean age (<70/≥70 years). The pooled HRs were calculated with the use of STATA METAN [31]. The separate HRs reported in subgroups categorized by body mass index (BMI) were revaluated by combining the enrolled number and the death case under a different ALT level range with the use of STATA METAN [31]. Second, the combined HRs of all-cause, CV disease-related, cancer-related, and liver disease-related mortality were calculated between higher and lower ALT levels using the most fully adjusted covariates estimated in the younger and older population separately. Third, the dose-response HR of all-cause, CV disease-related, cancer-related, and liver disease-related mortality with 5 U/l of ALT increment in the two separate groups was calculated according to reported data (categories of ALT levels on median dose, number of cases and participants, and effect estimates with corresponding standard errors were required) using a method previously proposed [32], [33]. Median or mean values in each group were presented according to categories of ALT activity. When this information was not reported, the midpoint of the upper and lower boundaries (as the approximate medians) was assigned. When the highest category was open-ended, the lower end value of the category multiplied by 1.2 was assigned [33]. Pooled HRs of disease-specific deaths caused by 5 U/l of ALT increment were then estimated to further analyze the association between ALT activity and disease-specific mortality in the two separate groups.

To explore the influence of age on the association between ALT levels and all-cause/disease-specific mortality, meta-regression analyses were performed with the METAREG command [34]. The robustness of findings with respect to different assumptions was estimated by conducting a sensitivity analysis with METAINF command to search for outliers that might be the main origin of the heterogeneity in the overall analysis. Outliers identified were omitted and the impact was re-evaluated in the residual studies.

Furthermore, subgroup analysis was performed on enrolled studies divided by gender, ethnicity, follow-up duration, sample size, comparisons, observational indicator, previous use of statin medication, whether mainly (>95%) in the self-defined normal range of ALT levels, whether adjusted for alcohol intake, and MetS components (four or more MetS components adjusted according to the NCEP-ATP-III criteria [35]) as covariates in younger and older subgroups (classified by mean age <70 years/≥70 years, respectively). All the subgroup analysis aimed to explore the sources of potential variability in examining the association between ALT level and all-cause/disease-specific mortality.

The heterogeneity among studies was evaluated by the Chi-square-based Q test and I^2^ test. Values of 25%, 50%, and 75% value for I^2^ represented low, moderate, and high heterogeneity, respectively [36]. If there was no significant heterogeneity (*P*-value >0.05 and I^2^<50%), the fixed-effect model was chosen to estimate the summary HR and 95% CI. Otherwise, the random-effect [37] model was used. Finally, Begg's funnel plot and Egger's test [38], [39] were used to assess potential publication bias with the METABIAS command [40] in STATA. A *P*-value >0.05 was considered insignificance. The meta-analysis was performed using Stata 12.0 software (Stata, College Station, TX, USA).

## Results

### Literature retrieval

The flow chart of enrolled studies for systematic review and meta-analysis is shown in Figure S1. A total of 4061 studies were retrieved from three databases (PubMed, Embase, and ISI) after the exclusion of 417 duplications. Among the 4061 studies, 4034 were removed by screening the titles and abstracts. The remaining 27 publications were independently screened through two authors (Zhengtao Liu and Shuping Que). Ten papers were finally included in the review (Cohen's Kappa = 0.724). The process and reasons for exclusion are shown in Figure S1.

### Characteristics of the included studies

Twelve prospective cohort studies, included in ten literatures involving 206,678 participants and 16,249 all-cause deaths in the follow-up duration, were enrolled in the systematic review and meta-analysis (Table 1). Among the twelve studies, three were performed in Asia, seven were performed in Europe, and three were performed in the USA. The mean age of participants ranged from 42 to 85 years and the durations of follow-up ranged from 2 to 14 years. Seven studies reported on the association between ALT level and specific mortality caused by CV, cancer, and liver disease [3], [11], [13], [14]. CV disease-related mortality was the major cause of mortality, with 4517 deaths in the follow-up (accounting for 27.8% of the overall mortality), followed by mortality due to cancer (3767 deaths, 22.6% of the overall mortality), and deaths due to liver disease (558 deaths, 3.43% of the overall mortality). Most studies enrolled both men and women, except for a German [8] and Scottish study (WOSCOPS) [13] that only focused on men). Seven of the twelve studies defined their normal range of ALT levels (from 19 to 55 U/l). The percentage of participants within a self-defined normal range of ALT values ranged from 85.6% to 96% in respective studies. One publication excluded those infected with the hepatitis B virus (HBV) or hepatitis C virus (HCV) infectors [11]. Another publication (including two data clusters) excluded the ALT outlier subjects (higher than three times the cut-off value) [13]. The majority of enrolled studies adopted the HR as an observational indicator, except for two studies that applied RR as an observational indicator [3], [8]. All the studies evaluated HRs (or RRs) and 95% CIs of mortality between participants with higher and lower ALT activity after adjusting confounding covariates. HRs evaluated in specific subgroups, classified by age or genders, were also presented if provided in enrolled literatures (Table 1). One Japanese study [4] reported the HR in a population categorized by median BMI and the HR was revaluated in the whole population (Table 1).

**Table 1:** Characteristics of enrolled cohort prospective studies included in meta-analysis.

First author and publication year	Country	Population characteristic	Definition of normal ALT level (U/L)	Enrolled subjects (M/F)	Age (range, mean±SD, years)	Outcome (events number) gender/age (years)	Follow up (years)	Comparison (U/L)	HR (95%CI)^a^	Adjusted covariates
Arndt et al [8]	Germany	Construction	Not	7858	42.8	All-cause (163)	5	>22/≤22	1.3(0.9–1.9)	Age, nationality,
(1998)		workers	referred	(7858/0)	(25-64)					occupational group,
										smoking status, BMI,
										alcohol consumption
Kim et al [3]	Korea	Insured	35–40	142055	M:	All-cause(3392)M	8	M:≥100/<20	5.2(4.2–6.4)	Age, BMI, smoking
(2004)		workers in	93.5% of	(94533	44.8±6.7	All-cause(394)F		F: ≥50/<20	1.2(0.5–3)	status, alcohol
		Korea Medical	subjects	/47522)	F:	CV-disease(624)M		M:≥100/<20	2.9(1.5–5.6)	consumption, plasma
		Insurance	within		42.0±6.0	CV-disease(52)F		F: 30–39/<20	1.3(0.4–4.1)	glucose, serum total
		Corporation	normal			Cancer(1514)M		M:≥100/<20	6.2(4.2–8.3)	cholesterol, blood
			range			Cancer(235)F		F: > = 100/<20	0.9(0.2–3.5)	pressure, family
						Liver disease(501)M		M:≥100/<20	59(43.4–80.1)	history of liver
						Liver disease(23)F		F: ≥50/<20	21.5(5.3–81.6)	disease
Elinav et al [15]	Israel	Community	< 40 for	455	70	All-cause(146)	12	>median	0.67	Sex, physical activity,
(2006)		residents	men,	(245/210)				/≤median	(0.46–0.93)	health perception,
			<30 for					Median:13 in		diabetes mellitus,
			women,					men/11 in		IHD, malignancy, CRF,
			96% of the					women		anemia, smoking
			subjects							
			within							
			normal							
			range							
Nalamura et al [4]	Japan	Community	Not	4524	40–69	All-cause (214)	10	Below median	8.11	Age, sex, BMI,
(2006)		residents	referred	(1531/2593)	54.3±8.1			BMI^b^: ≥50/<20	(3.16–20.82)	smoking habit,
								Up than		drinking habit, SBP,
								median BMI:	1.38	medication for
								≥50/<20	(0.34–5.63)	hypertension, serum
								Whole		total cholesterol,
								population:	3.72	history of diabetes
								(including	(1.95–7.10)	mellitus
								people with		
								lower and		
								higher BMI)		
Schindhelm et al	Nether-	Community	Not	1439	50–75	All-cause (174)	10	T3/T1	1.10	Age, sex,
[9] (2007)	lands	residents	referred	(788/651)	60.9±7.2			T3:26(21–143)	(0.77–1.61)	alcohol-intake,
								T1:12(1-14)		smoking, physical
										activity, waist,
										triglycerides, SBP,
										fasting glucose,
										HDL-C
Ruhl et al [11]	USA	Community	≤30 for	14950	Normal	All-cause(1205)M	8.8	Elevated ALT	1.1(0.62–1.9)	Age, sex,
(2009)		residents with	men,	(6953/7997)	ALT	All-cause(984)F		/normal ALT	1.2(0.82–1.8)	race-ethnicity, BMI,
		exclusion of	≤19 for		group:	CV-disease(379)M		(Normal ALT	0.38(0.12–1.2)	waist-to-hip ratio,
		HBV and HCV	women,		45.3±0.50	CV-disease(286)F		definition:	1.2(0.64–2.2)	glucose status, total
		infectors	85.6% of		elevated	Cancer(291)M		M≤30, F≤19)	1.3(0.66–2.6)	cholesterol, HDL-C,
			subjects		ALT group:	Cancer(208)F			0.9(0.43–1.9)	SBP, DBP, smoking,
			within		42.1±0.60	Liver disease (22)M			8.8(2.0–38.7)	alcohol, caffeine,
			normal			Liver disease (12)F			8.7(1.03–74)	physical activity, CRP,
			range							transferrin
										saturation, education
Hovinen et al [17]	Finland	Community	Not	397	M: 75–91	All-cause (127)	5.8	M:≥21/<21	0.45	M: BMI, charlson
(2010)		residents	referred	(138/259)					(0.24–0.86)	comorbidity index,
										mini-mental state
										examination, peak
										expiratory flow,
										smoking, alcohol use,
										hemoglobin, glucose
					F: 75–90			F: ≥19/<19	0.62 (0.39–1)	F: Charlson
										comorbidity index,
										statin use, diabetes
										mellitus, DBP,
										heoglobin, LDL-C,
										HDL-C
Ford et al-	Scotland	Participants in a	≤55 largely	6595	45–64	All-cause(1293)	4.9	Q4/Q1	0.86	Treatment allocation,
WOSCOPS		clinical trail of	within	(6595/0)	55.24–90			(>27/≤17)	(0.73–1.01)	age, history of
[13](2011)		pravastatin	normal							angina, history of
		with exclusion	range			CV-disease (377)			0.87	diabetes and
		of ALT outlier							(0.64–1.18)	hypertension,
		(≥165 IU/L)								smoking status, BMI,
						Cancer (532)			0.82	SBP, DBP, HDL-C,
									(0.63–1.07)	LDL-C,
										log(triglycerides),
										glucose, nitrate use,
										socioeconomic
										deprivation, alcohol
										use
Ford et al-	Scotland,	Participants in a	≤55 largely	5804	70–82	All-cause (604)	3.2	Q4/Q1	0.64	Country, treatment
PROSPER [13]	Ireland,	clinical trail of	within	(2803/3001)	75.3±3.3			(>22/≤14)	(0.5–0.81)	allocation, age, sex,
(2011)	Netherland	pravastatin	normal							current smoker and
		with exclusion	range			CV-disease (216)			0.58	histories of diabetes,
		of ALT outlier							(0.39–0.87)	hypertension
		(≥165 IU/L)								(components of
						Cancer (206)			0.68	vascular disease,
									(0.45–1.03)	BMI, SBP, DBP,
										HDL-C, LDL-C,
										log(triglycerides),
										glucose, alcohol
										consumption
Ford et al-	Nether-	Community	≤45 largely	561	85	All-cause (451)	2	Q4/Q1	0.66	Sex, BMI,
Leiden 85-plus	lands	residents	within	(188/373)				(>27/≤17)	(0.5–0.87)	hypertension, SBP,
[13] (2011)			normal							DBP, HDL-C, LDL-C,
			range			CV-disease (48)			0.8	triglycerides, CRP,
									(0.34–1.88)	diabetes, HbA1c,
										history of vascular
						Cancer (78)			0.63	disease
									(0.32–1.21)	
Schooling et al	USA	Community	Not	16854	T1^c^:	All-cause(2199)M	13.2	M:T3/T1	0.89(0.70–1.12)	Age, gender,
[14] (2012)		residents	referred	(7888/8966)	49.8±22.9	All-cause(1906)F		F:T3/T1	0.99(0.85–1.14)	race/ethnicity,
					T2^d^:	All-cause(266)<50 yr		<50 yr: T3/T1	1.15(0.67–1.99)	education, smoking
					46.9±20.1	All-cause(1110)50–75 yr		50–75 yr:T3/T1	0.96(0.76–1.22)	status, alcohol use
					T3^e^:	All-cause(2729)>75 yr		>75 yr: T3/T1	0.87(0.75–1.01)	
					43.5±17.1	CV-disease(974)M		M:T3/T1	0.86(0.66–1.11)	
						CV-disease(889)F		F:T3/T1	0.93(0.73–1.17)	
						CV-disease(58)<50 yr		<50 yr: T3/T1	1.66(0.64–4.31)	
						CV-disease(436)50–75 yr		50–75 yr:T3/T1	1.02(0.68–1.53)	
						CV-disease(1369)>75 yr		>75 yr: T3/T1	0.81(0.68–0.96)	
Koehler et al [18]	Nether-	Community	< 40 for	5186	70.3 ± 9.1	All-cause (2997)	14	P95/P25	0.92	Age, sex, education,
(2013)	land	residents	men,	(3195/1991)					(0.76–1.11)	smoking status,
			<30 for							alcohol intake,
			women,			CV-disease (672)			0.87	hypertension,
			94.8% of						(0.57–1.29)	diabetes mellitus,
			subjects							BMI, total
			within			Cancer (703)			1.05	cholesterol levels
			normal						(0.74–1.50)	
			range							

a two studies [3], [8] only provided RR as observational indicator. RR was treated as HR unless specific notification.

b the median BMI in study was 22.7 kg/m^2^.

c T1 represented the subjects with lowest category of ALT value (≤13 U/l for men and ≤9 U/l for women)

d T2 represented the subjects with middle category of ALT value (13–21 U/l for men and 9–15 U/l for women)

e T3 represented the subjects with highest category of ALT value (≥21 U/l for men and ≥15 U/l for women)

Abbreviations: ALT: alanine aminotransferase; BMI: body mass index; CI: confidence interval; CRF: chronic renal failure; CRP: C-reactive protein; CV: cardiovascular; DBP: diastolic blood pressure; F: female; HbA1c: glycated haemoglobin; HBV: hepatitis B virus; HCV: hepatitis B virus; HDL-C: high-density lipoprotein cholesterol; HR: hazard ratio; IHD: ischemic heart disease; LDL-C: low density lipoprotein cholesterol; M: male; P: percentile; Q: quartile; SBP: systolic blood pressure; SD: Standard deviation; T: tertile; U/l: units per liter; yr: year.

### Quality assessment results

All of the prospective studies were of high quality (NOS score >6). The average NOS score of the studies overall was 7.58. The quality assessment scores by NOS are shown in Table S2.

### Meta-analysis

**Figure 1:**
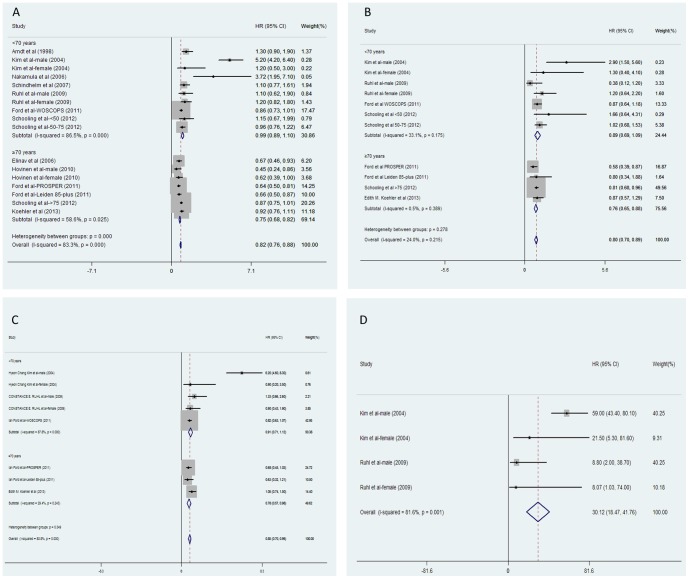
Meta-analysis of comparing hazard ratio of mortality between the highest versus lowest category of ALT levels classified by age. A represented the ALT-all cause mortality association; B represented the ALT-CV related mortality association; C represented the ALT-cancer related mortality; D represented the ALT-liver disease related mortality. Abbreviations: ALT: alanine aminotransferase; HR: hazard ratio.

**Figure 2:**
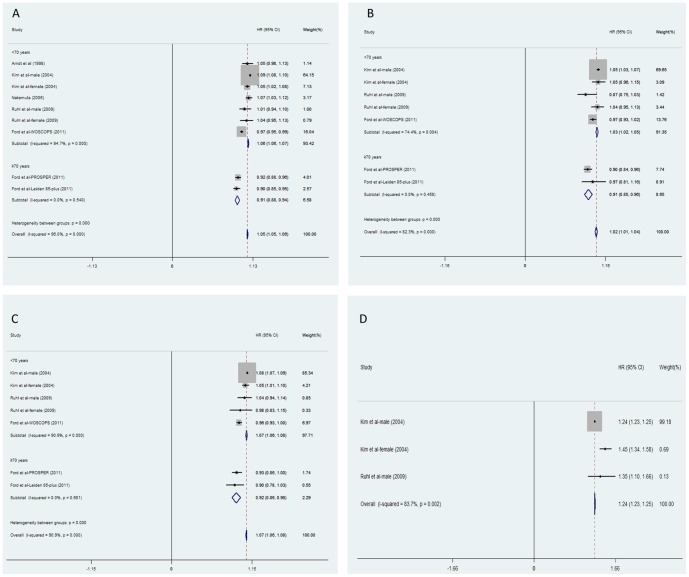
Meta-analysis of comparing hazard ratio of mortality with 5 U/l of ALT increment classified by age. A represented the ALT-all cause mortality association; B represented the ALT-CV related mortality association; C represented the ALT-cancer related mortality; D represented the ALT-liver disease related mortality. Abbreviations: ALT: alanine aminotransferase; HR: hazard ratio.

**Figure 3:**
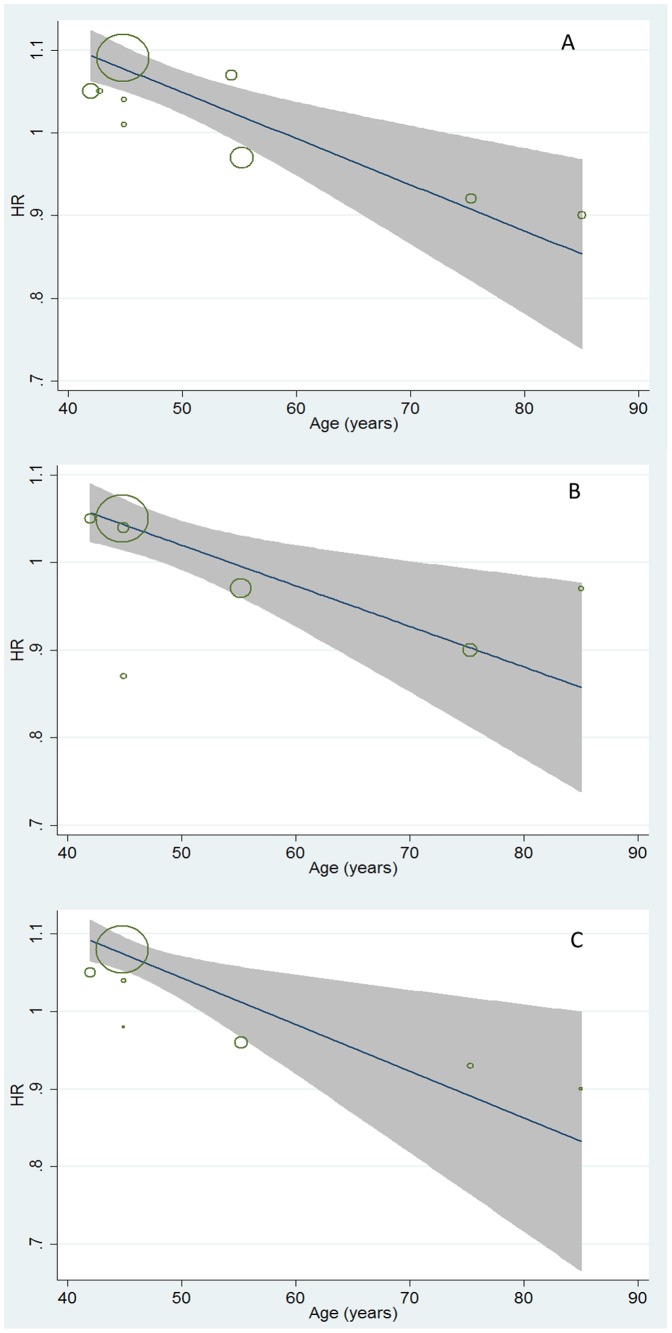
Meta- regression analyses on ALT-mortality association by age. A represented the impact of age on ALT-all cause mortality association (coefficient: 0.996, 95%CI: 0.994–0.998); B represented the impact of age on ALT-CV related mortality association (coefficient: 0.996, 95%CI: 0.992–0.998); C represented the impact of age on ALT-cancer related mortality (coefficient: 0.996, 95%CI: 0.992–0.998).

According to age and gender, seventeen, eleven, eight and four data clusters were extracted to evaluated the pooled HRs of all-cause, CV disease-related, cancer-related, and liver disease-related mortality, compared between participants with higher and lower ALT levels respectively (in overall, younger, and older subgroups; Figure 1). With regard to the HRs compared between participants with higher and lower ALT levels, the heterogeneities of HRs decreased from all-cause to disease-specific mortality. In addition, the heterogeneities of HRs in the younger population were larger than the older population (Figure 1). When pooling the dose-response HRs of all-cause, CV disease-related, cancer-related, and liver disease-related mortality per 5 U/l of ALT increment (Figure 2), heterogeneity significantly decreased in the older subgroup. However, the heterogeneity was still significant in the younger population. In the older population, negative associations were observed between ALT increment and all-cause, CV disease-related, cancer-related mortality (HR: 0.91, 95% CI: 0.88–0.94, for all-cause mortality; HR: 0.91, 95% CI: 0.85–0.96, for CV disease-related mortality; HR: 0.92, 95% CI: 0.86–0.98, for cancer-related mortality). Pooled HRs in the older population were much lower than HRs in younger people (all heterogeneity between subgroups *P*<0.001, Figure 3). In younger adults, moderately elevated HRs of all-cause mortality (HR: 1.06, 95% CI: 1.06–1.07), CV-related mortality (HR: 1.03, 95% CI: 1.02–1.05), and cancer-related mortality (HR: 1.07, 95% CI: 1.06–1.08) per 5 U/l of ALT increment were observed with significant heterogeneity (Figure 2). Notably, high HRs of liver-disease related mortality per 5 U/l of ALT elevation were observed in every referred study (all in the younger population). An approximate increase of 24% in liver-disease related mortality with 5 U/l of ALT increment was observed (HR: 1.24, 95% CI: 1.23–1.25; Figure 2).

Furthermore, the quantitative impacts of age on the association between ALT variation and mortality (from all-cause, CV disease-related, and cancer-related) were evaluated by meta-regression analysis (Figure 3). Moderate but consistent inverse effects of age on HRs of all-cause, CV disease-related, and cancer-related mortality caused by ALT increment were observed. A decrease of approximately 4‰ on dose-response HRs of mortality related to all-cause (95% CI: 0.994–0.998), CV-disease (95% CI: 0.992–0.998), and cancer (95% CI: 0.994–0.998) followed with one year's age increment were observed with significant trends (all *P*<0.05, Figure 3).

### Sensitivity and subgroup analyses

**Figure 4:**
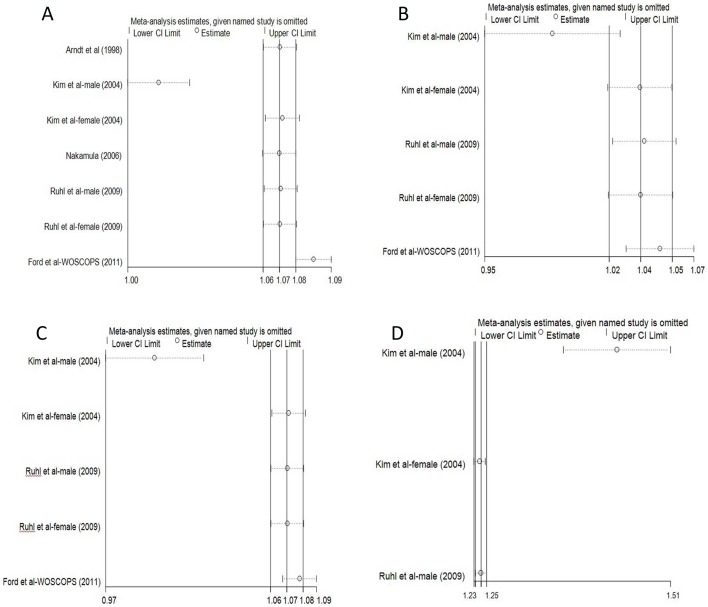
Sensitivity analyses by stepwise omitting each study at a time in younger adults. A represented the re-evaluation of ALT-all cause mortality association after omitting each study; B represented the re-evaluation of ALT-CV related mortality association after omitting each study; C represented the re-evaluation of ALT-cancer related mortality association after omitting each study; D represented the re-evaluation of ALT-liver disease related mortality after omitting each study.

**Figure 5:**
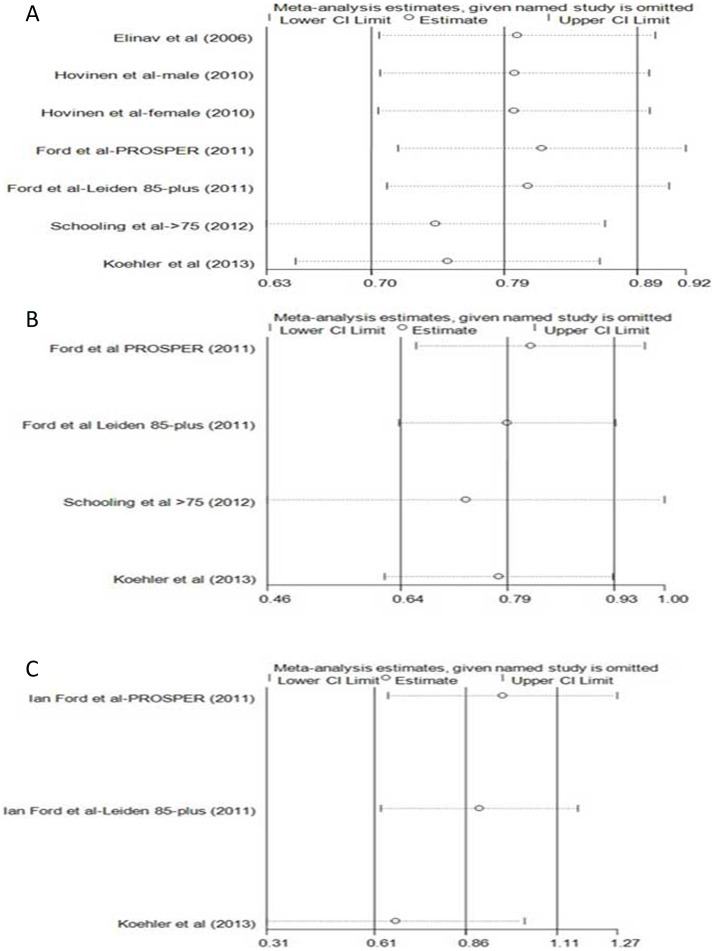
Sensitivity analyses by stepwise omitting each study at a time in older subjects. A represented the re-evaluation of ALT-all cause mortality association after omitting each study; B represented the re-evaluation of ALT-CV related mortality association after omitting each study; C represented the re-evaluation of ALT-cancer related mortality after omitting each study.

Because high heterogeneity was present in the overall analysis, sensitivity analyses were conducted by stepwise omitting one study and re-evaluating the summary HRs of relative mortality on the residual studies to estimate the impact of a single study on combined results classified by age (Figure 4, Figure 5). In younger adults, the data clusters from Korean males [3] and the Scottish population with statin medication [13] were the outliers that caused the HRs of all-cause, CV disease-related, and cancer-related mortality deviated from the middle. Meanwhile, results reported in Korean males led to outlying liver-disease related mortality in the overall analysis (Figure 4). For older people, the American and Dutch studies [14], [18] also influenced the overall results; however, the impacts were lower than the outliers in younger adults (Figure 5). These outliers also contributed to the heterogeneity in subgroup analysis.

In the following sensitivity analysis, the pooled HRs were revaluated after excluding the outliers by age (Table 2). The heterogeneity decreased to insignificance in most ALT-mortality associations, except for the ALT-CV-disease mortality in the younger population (Table 2). The pooled HRs decreased further after excluding the outliers, except for the pooled HR of liver-disease related mortality caused by ALT elevation in the younger subgroup (Table 2). ALT activity lost its significant association with CV disease-related mortality after excluding the outliers in the younger population (HR: 1.01, 95% CI: 0.95–1.07; Table 2).

**Table 2:** Sensitivity analysis on pooled HR of mortality after excluding the outliers classified by age.

Group	Number of data clusters (n)	HR^a^(95%CI)	I^2^ (%)	*P*
Younger subgroup (mean age < 70 years)				
All-cause mortality				
Studied with no exclusion	7	1.06(1.06–1.07)	94.7	<0.001
Studies except the Korean outlier [3]	6	1.01(0.99–1.02)	83.5	<0.001
Studies except the Korean and Scottish outliers [3], [13]	5	1.05(1.03–1.07)	0	0.781
CV mortality				
Studies with no exclusion	5	1.03(1.02–1.05)	74.4	0.004
Studies except the Korean outlier [3]	4	0.99(0.95–1.02)	52.0	0.100
Studies except the Korean and Scottish outliers [3], [13]	3	1.01(0.95–1.07)	59.9	0.085
Cancer mortality				
Studies with no exclusion	5	1.07(1.06–1.08)	90.9	<0.001
Studies except the Korean outlier [3]	4	1.00(0.97–1.02)	71.1	0.016
Studies except the Korean and Scottish outliers [3], [13]	3	1.04(1.00–1.08)	0	0.709
Liver disease mortality				
Studies with no exclusion	3	1.24(1.23–1.25)	83.7	0.002
Studies except the Korean outlier [3]	2	1.43(1.32–1.55)	0	0.520
Older subgroup (mean age≥70 years)				
All-cause mortality				
Studied with no exclusion	7	0.75(0.68–0.82)	58.6	0.025
Studies except the American and Dutch outliers [14], [18]	5	0.63(0.54–0.73)	0	0.822
CV mortality				
Studies with no exclusion	4	0.75(0.63–0.87)	24.4	0.266
Studies except the American and Dutch outliers [14], [18]	2	0.60(0.37–0.83)	0	0.593

a HR calculated in younger subgroup was the dose-response evaluation assessed per 5 U/l of ALT increment;

HR calculated in older subgroup was the evaluation compared between higher and lower ALT categories.

Abbreviations: CI: confidence interval; CV: cardiovascular; HR: hazard ratio.

In addition, we attempted to evaluate the potential source of heterogeneity by subgroup analyses classified by age (mean age <70/≥70 years). Potential covariates that might contribute to heterogeneity were estimated in the association between ALT activity and all-cause/cause specific mortality in subgroup analysis (Table 3, Table 4). In the younger population, the pooled HR of all-cause, CV disease-related, and cancer-related mortality was unstable and inconsistent in subgroup analysis classified by ethnicity, sample size, and statin medication (Table 3). Notably, the HRs of all-cause, CV disease-related, and cancer-related mortality were much higher in Asians and populations without statin medication. The pooled RRs of all-cause, CV disease-related, and cancer-related mortality were much higher than the pooled HRs (all *P*≤0.001, Table 3). In the older population, the pooled HRs were more stable than younger adults. A significant and negative association was still observed in separate subgroups, except in the subgroup with a smaller sample size (<1000), without adjusting for alcohol intake (on ALT-CV disease-related mortality association, Table 4). The significant heterogeneity on HRs of all-cause mortality between subgroups classified by potential confounders might originate from the outliers [14], [18]. In addition, the inter-group heterogeneities decreased to insignificance when the subgroup analysis was performed after excluding the outliers (all *P*>0.05, data not shown).

**Table 3:** Subgroup analysis on HRs of mortality in younger population (mean age < 70 years).

Group	Number of data clusters (n)	HR^a^ (95%CI)	I^2^ (%)	*P* value for heterogeneity in subgroups	*P* value for heterogeneity between subgroups
All-cause mortality					
Gender					
Male	4	1.07(1.06–1.07)	97.2	<0.001	
Female	2	1.05(1.02–1.08)	0	0.836	0.290
Race/ethnicity					
Asian	3	1.09(1.08–1.09)	69.7	0.037	
Non-Asian	4	0.98(0.96–1.00)	54.2	0.088	<0.001
Follow-up (mean year)					
<8	4	1.06(1.06–1.07)	97.3	<0.001	
≥8	3	1.05(1.02–1.09)	0	0.419	0.545
Sample size (n)					
<10000	5	0.99(0.98–1.01)	79.8	0.001	
≥10000	2	1.09(1.08–1.10)	83.7	0.013	<0.001
Statin medication					
No	6	1.08(1.07–1.09)	57.1	0.040	
Yes	1	0.97(0.95–0.99)	none	none	<0.001
Adjustments of MetS components^b^					
No	2	1.06(1.03–1.10)	0	0.654	
Yes	5	1.06(1.06–1.07)	96.5	0	0.966
Observational indicator					
RR	3	1.09(1.08–1.09)	71.5	0.030	
HR	4	0.99(0.97–1.01)	82.8	0.001	<0.001
CV-mortality					
Gender					
Male	3	1.03(1.02–1.05)	87.1	<0.001	
Female	2	1.04(0.98–1.11)	0	0.881	0.757
Race/ethnicity					
Asian	2	1.05(1.03–1.07)	0	1	
Non-Asian	3	0.98(0.94–1.01)	52.5	0.122	0.001
Follow-up (mean year)					
<8	3	1.04(1.02–1.06)	80.4	0.006	
≥8	2	0.99(0.91–1.07)	75.0	0.045	0.237
Sample size (n)					
<10000	3	0.98(0.94–1.01)	52.5	0.122	
≥10000	2	1.05(1.03–1.07)	0	1	0.001
Statin medication					
No	4	1.05(1.03–1.07)	52.0	0.100	
Yes	1	0.97(0.93–1.01)	None	None	0.002
Observational indicator					
RR	2	1.05(1.03–1.07)	0	1	
HR	3	0.97(0.93–1.01)	52.5	0.122	0.001
Cancer-mortality					
Gender					
Male	3	1.07(1.06–1.08)	95.3	0	
Female	2	1.04(1.00–1.09)	0	0.409	0.255
Race/ethnicity					
Asian	2	1.08(1.07–1.09)	38.5	0.202	
Non-Asian	3	0.97(0.94–1.00)	9.4	0.331	<0.001
Follow-up (mean year)					
<8	3	1.07(1.06–1.08)	95.3	<0.001	
≥8	2	1.02(0.94–1.11)	0	0.533	0.281
Sample size (n)					
<10000	3	0.97 (0.94, 1.00)	9.4	0.331	
≥10000	2	1.08 (1.07, 1.09)	38.5	0.202	<0.001
Statin medication					
No	4	1.08 (1.07, 1.09)	17.5	0.304	
Yes	1	0.96 (0.92, 1.00)	None	None	<0.001
Observational indicator					
RR	2	1.08 (1.07, 1.09)	38.5	0.202	
HR	3	0.97 (0.94, 1.00)	9.4	0.331	<0.001

a the HR of mortality was dose-responded per 5 U/l of ALT increment.

b the adjusted MetS components were defined by NCEP-ATP-III criteria [35]; “Yes” represented four or more MetS covariates adjusted; “No” represented less than four MetS covariates adjusted.

Abbreviations: CI: confidence interval; CV: cardiovascular; HR: hazard ratio.

**Table 4:** Subgroup analysis on HRs of mortality in older population (mean age≥70 years).

Group	Number of data clusters(n)	HR^a^(95%CI)	I^2^ (%)	*P* value for heterogeneity in subgroups	*P* value for heterogeneity between subgroups
All-cause mortality					
Comparison^b^					
Up than median/below median ALT	3	0.60(0.44–0.76)	0	0.534	
Highest/lowest ALT	4	0.78(0.71–0.86)	66.8	0.029	0.041
Follow-up (mean year)					
<10	4	0.62(0.52–0.73)	0	0.706	
≥10	3	0.85(0.76–0.95)	32.4	0.228	0.001
Sample size (n)					
<1000	4	0.62(0.50–0.75)	0	0.681	
≥1000	3	0.81(0.72–0.90)	71.3	0.031	0.014
Statin medication					
No	6	0.78(0.70–0.85)	58.9	0.033	
Yes	1	0.64(0.48–0.80)	none	none	0.127
Adjustments of MetS components^c^					
No	4	0.76(0.66–0.86)	62.3	0.047	
Yes	3	0.73(0.64–0.83)	68.6	0.041	0.702
Adjustment of alcohol intake					
No	3	0.66(0.52–0.79)	0	0.966	
Yes	4	0.78(0.70–0.87)	74.5	0.008	0.105
Subjects mainly in normal range^d^					
No	4	0.82(0.73–0.92)	66.5	0.030	
Yes	3	0.65(0.55–0.76)	0	0.974	0.019
CV-mortality					
Follow-up (mean year)					
<10	2	0.60(0.37–0.83)	0	0.593	
≥10	2	0.82(0.69–0.95)	0	0.761	0.104
Sample size (n)					
<1000	1	0.80(0.30–1.57)	none	none	
≥1000	3	0.76(0.65–0.88)	33.5	0.223	0.927
Statin medication					
No	3	0.82(0.69–0.95)	0	0.954	
Yes	1	0.58(0.34–0.82)	none	none	0.088
Adjustments of MetS components^c^					
No	1	0.81(0.67–0.95)	none	none	
Yes	3	0.68(0.48–0.87)	0	0.401	0.276
Adjustment of alcohol intake					
No	1	0.80(0.34–1.88)	none	none	
Yes	3	0.76(0.65–0.88)	33.5	0.223	0.927
Subjects mainly in normal range^d^					
No	2	0.82(0.69–0.95)	0	0.761	
Yes	2	0.60(0.37–0.83)	0	0.593	0.104

a HR was evaluated between higher and lower ALT categories.

b “Up than median/below median ALT” represented comparison between population with ALT value higher than median level and population with ALT value below the median level (ALT level was continuous); “Highest/lowest ALT” represented comparison between population with ALT value in the highest category and population with ALT value in the lowest category (ALT level was incontinuous).

c the adjusted MetS components were defined by NCEP-ATP-III criteria [35]; “Yes” represented four or more MetS covariates adjusted; “No” represented less than four MetS covariates adjusted.

d “No” represented the study not defined the “normal range” of ALT level or ≤95% of subjects were in the self-defined “normal range ” of ALT level; “Yes” represented the study defined the “normal range” of ALT level and >95% (or large part) of subjects were in the self-defined “normal range” of ALT level.

Abbreviations: ALT: alanine aminotransferase; CI: confidence interval; CV: cardiovascular; HR: hazard ratio.

### Assessment of publication bias

**Figure 6:**
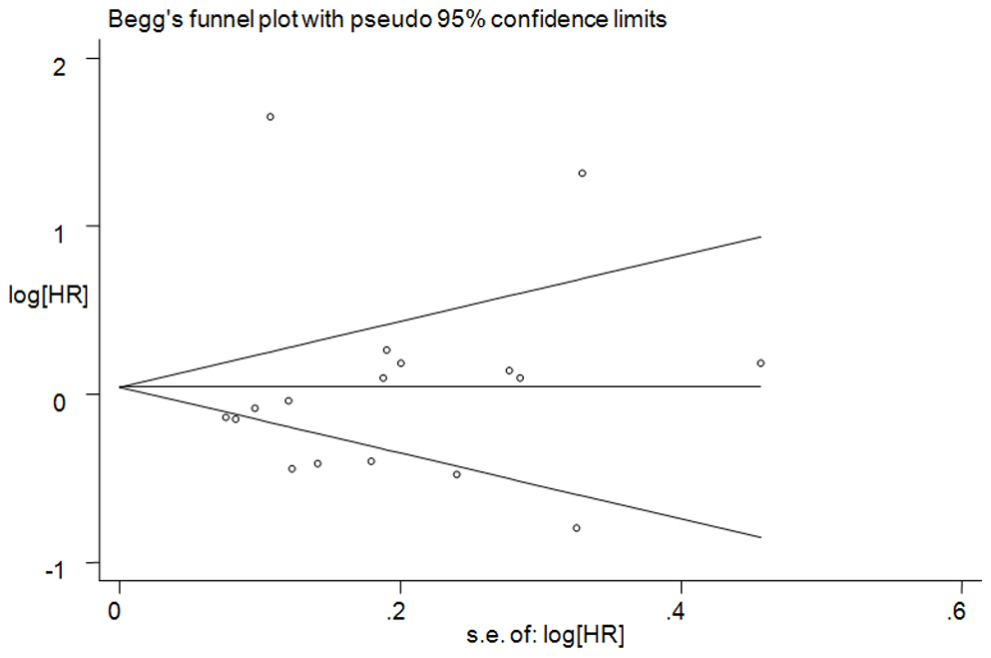
Begg's funnel plot analysis of publication bias. Egger's test: *P* = 0.936. Abbreviations: HR: hazard ratio.

To assess the publication bias, Begg's funnel plot (Figure 6) and Egger's test were used. No significant publication bias was observed (Egger's *P* = 0.936; Figure 6).

## Discussion

This is the first systematic review and meta-analysis to assess the complex association between ALT activities and ensuing all-cause/disease-specific (including CV disease, cancer, and liver-disease) mortality in prospective cohort studies. Twelve studies were examined and included a total of 206,678 participants and 16,249 deaths. In all the enrolled studies, age was an independent covariate influencing the dose-response association between ALT activity and all-cause, CV disease-related, and cancer-related mortality with a moderate but consistent impact in meta-regression analysis (Figure 3). An approximate decrease of 4‰ in dose-response HRs of mortality caused by ALT elevation was observed followed with one year's increment (all *P*<0.05). Hence, the enrolled studies were divided into two subgroups by mean age (<70/≥70 years). In the younger population, the HR of mortality from liver disease was 1.24 (95% CI: 1.23–1.25) with 5 U/l of ALT increment (Figure 2D). Conversely, an approximate decrease of 8–9% in all-cause, CV disease-related, and cancer-related mortality was observed, associated with a 5 U/l ALT elevation with low heterogeneity in older population (Figure 2). Overall, the heterogeneity of pooled HRs was more obvious in younger adults (Figure 1, Figure 2). Compared to the older population, significantly higher dose-response HRs for all-cause, CV disease-related, and cancer-related mortality was observed in younger subgroups (all *P*<0.01; Figure 2).

In younger adults, the ALT activity was weakly, unstably, and inconsistently associated with all-cause, CV disease-related, and cancer-related mortality, even after excluding the outliers. The inclusion of Korean male participants [3] significantly increased the pooled results, possibly because of the relatively higher occurrence of liver-disease related deaths, which might be linked to the high prevalence of HBV infection observed in that specific population [41]. Otherwise, the population using statin medication in the Scottish study [13] might also influence and cause a decrease in the pooled HRs. However, these impacts were limited and the pooled HRs were still low after excluding the outliers. In previous studies, ALT activity was considered useful in predicting the overall health and mortality risk [1], [26]. Through meta-analysis on all relative studies, the associations between ALT activity and all-cause/disease-related (including CV-disease and cancer) mortality were inconsistent, susceptible to many covariates and weak in synthesizing the dose-response HRs from different studies. Accordingly, the results indicated that ALT was not an available predictor when using all-cause or non-hepatic death as the observational endpoints in epidemiological surveys of the younger population. However, further investigations are needed.

In contrast, the ALT activity was strongly linked to liver disease-related death, even after excluding the participants with viral hepatitis [11]. ALT activity is more hepatic-specific than other liver enzymes (e.g., aspartate aminotransferase [AST], gamma-glutamyltransferase [GGT]) and sensitive in predicting liver disease [2], [42]. ALT elevation is indicative of apparent or latent liver disease (such as non-alcoholic fatty liver disease (NAFLD), alcoholic liver disease (ALD) and viral hepatitis infection [43]–[45]), which might cause more deaths in the general population. However, the significant risk of liver disease-related mortality caused by ALT elevation was reported to exhibit a huge discrimination due to distinctions in enrollment and comparison (Figure 1D). In this study, the ALT elevation was standardized and an approximate increase of 43% in liver-disease related mortality was observed to follow the same extent of ALT elevation (per 5 U/l) after excluding the outliers (results from the Korean male population [3] with a high prevalence of HBV infection [41]). This indicated that ALT elevation is closely associated with liver disease-related mortality.

With regard to the older samples, significant negative associations were observed between ALT activity and all-cause/disease-specific (including CV disease, cancer) mortality after synthesizing the dose-response HRs (Figure 2). An approximate 8–9% decrease in all-cause, CV disease-related and cancer-related mortality followed with 5 U/l of ALT increment (mostly in the normal range) was observed after pooling the reported HRs. Furthermore, these inverse associations were still significant even after adjusting the MetS components (Table 4). This phenomenon was first observed by Elinav et al in 2006 [15] and confirmed by several research studies [13], [14], [18], [19] thereafter. The mechanism responsible for the inverse association between ALT level and mortality remains uncertain. Recently, an ultra low ALT level was considered a biomarker of an exaggerated hepatic aging process [15], [22], including a reduction in liver size, blood flow, regeneration, and alexipharmic functions in hepatic aspects [24], [46]. It was also implied in frailty and poor nutritional conditions [19], which might induce more deaths. Elevated ALT level is strongly associated with the increasing prevalence of MetS, which is linked to higher mortality in older population [47], [48]. Accordingly, elevated ALT activity is theoretically related to a higher mortality. However, the inverse association between ALT activity and mortality was still statistically significant after adjusting for the MetS covariates (Table 4), indicating that the ALT level might be more representative of aging and frailty in the older population.

Notably, two studies [14], [18] significantly influenced the pooled results and introduced more heterogeneity in the older population. One HR was calculated between the subjects with upper tertile and the lower tertile of ALT category, and the extremely high ALT was not excluded [14]. Another mortality comparison was performed between the participants in the top 5% (>33 U/l higher than the health range previously recommended before [49], [50]) and the bottom 5% category of ALT levels [18]. In general, the inclusion of participants with ALT outliers in these two studies might confound the pooled results. Otherwise, four studies [13], [15], [18] of older populations specifically defined the normal range of ALT value (Table 1). In addition, the decreased pooled HR and heterogeneity in subgroups with participants mainly in the self-defined normal range of ALT levels (Table 4) implied that lower ALT levels might be more associated with higher mortality in older populations when making comparisons based on a normal range based in the published literature. Recently, deep concerns have been raised on the association between extremely low ALT levels and higher mortality, especially in the older populations [25]. Similar to previous studies [13], [15], [17], results in this study confirmed the inverse association and quantitatively estimated the dose-response risk of all-cause mortality and disease-specific (CV disease and cancer) mortality in older adults (Figure 2). The upper limit of normal (ULN) values for ALT level has been defined in given population in prior population-based studies [49]–[52]. However, no study has focused on the lower limit of normal of ALT levels, and an appropriate age-specific lower limit for a normal ALT level is necessary to enhance the predictive value of ALT as a common biomarker used in the health care of older subjects. The inverse association between ALT level and mortality warrants further investigation.

Remarkably, age showed a crucial impact on the association between ALT level and the ensuing all-cause/disease-specific mortality (all *P*<0.05, Figure 3). The dose-response HRs of mortality caused by ALT elevation were quantitatively assessed, and a 4‰ decreases in these HRs were observed with moderate but consistent effects followed with one year's age increment (Figure 3). The mechanisms on how age affects the ALT-mortality association are not fully understood. One possibility is that ALT activity is more liver-specific and that ALT elevation is more indicative of death from apparent or latent liver disease, rather than all-cause mortality in younger adults (Figure 2D). Another explanation is that, with increasing age, decreases in ALT levels might be more representative of aging and frailty in overall health (independent of its traditional role in screening for liver function) [20], [21]. Similar to prior hypotheses [26], we speculate that the clinical significance of ALT activity is affected by age and that the meta-regression proved this impact (Figure 3).

Except for age, the inconsistence of covariates, including the characteristics of enrolled participants and the observational index, might also confound the ALT-mortality association and should be taken into consideration. ALT elevation is often associated with MetS, alcoholic intake, and medication [2], [43], [45], [53]–[55]. Inconsistence in these variables during enrollment might interfere and introduce biases on the final results. MetS is a series of metabolic disorders, including obesity, dyslipidemia, hyperglycemia, and hypertension, commonly defined by the NCEP-ATP-III criteria [35]. The ALT-mortality association might be influenced by these metabolic disorders in the MetS patients [56]. However, the trend did not reverse when subgroup analysis was conducted adjusting four or more MetS components as covariates in the younger and older population, indicating a limited impact of MetS status on the ALT-mortality association. In regard to alcohol intake, all the studies in the younger population adjusted for this variable. In studies with older participants, no significant differences were observed between studies with or without adjustments for alcohol intake (Table 4). The limited impacts of alcohol intake on the ALT-mortality association might be due to the lower alcohol intake amount and fewer heavy drinkers in the older population [57], [58].

Two studies reported the ALT-mortality association in participants with moderate statin medication [13], which might cause slight liver toxicity manifested through a minor ALT elevation [59]. The influence of statin use on the ALT-mortality association is not fully clear. In this study, the effects of statin medication on the ALT-mortality association were inconsistent in different age groups. In younger adults, the statin medication might be a protective co-factor that decreased the risk of mortality (Table 3). Therefore, the pooled HRs of all-cause/disease- specific mortality were re-evaluated after excluding the outlier study [13]. These effects were attenuated in the older population and the heterogeneities of pooled HRs were no longer influenced by the study that enrolled participants taking statin medication (Table 4). With respect to observational indices, two studies [3], [8] (both examining younger populations) adopted the RR instead of HR to evaluate the ALT-mortality association. Inferior to HR, the RR did not consider the “time to an event”, censored data, and may have caused a bias when pooled with HR [60]. Accordingly, a decreased heterogeneity of pooled HRs was observed in subgroup analysis divided by observational indicators (Table 3). However, the refined pooled HRs still implied the weak and unstable association between ALT level and all-cause/disease-specific mortality in younger population (Table 3).

GGT activity is another common liver enzyme activity also considered closely associated with all-cause and CV disease-related mortality in prior meta-analysis [61]. ALT activity is obviously inferior to GGT activity in predicting the mortality, especially in younger adults. However, the predictive value of GGT on CV-related mortality decreased and even lost significance in the older populations [62]–[64]. As a supplement, the predictive value of ALT activity in screening for long-term survival might remedy the deficiencies of GGT on risk assessment in older populations.

Our findings have strengths and applications for clinical practice. A qualified librarian helped with designing an extensive search strategy to decrease the omission of relevant references. In addition, the extremely low ALT activity within a normal range is also clinically relevant to all-cause and disease-related mortality in older people. This phenomenon indicates that not all patients with ALT measured within a normal range are safe and will alert clinicians to consider extremely low ALT value within a normal range more cautiously. The older population is a fast growing segment that consumes majority of health care resources [65]. The ALT measurement can aid in the prediction of long-term survival and aid clinicians in making full use of medical resources for cost effectiveness.

Besides the previous deficiencies mentioned, several limitations in this meta-analysis should be addressed. First, the HRs of ALT-related mortality in the older population were mainly obtained from the European populations [13], [17]. For this reason, our results may not generalize well across other populations, such as the East Asians with a higher burden of HBV infection [66]. More studies in non-European older populations are thus required to confirm the association. Second, in addition to the intrinsic difference between ALT measurement assays performed at different institutes, heterogeneity observed in this study may be due to many factors, including differences in participant enrollment, sample size, statin medication use, follow-up lengths, and statistics used for comparisons. The subgroup analysis could not explain all the sources of heterogeneity, even when outlier studies were excluded. Third, the normal range of ALT levels were self-defined and distinguished. The negative ALT-mortality association was primarily obvious in older populations mainly within a self-defined normal range of ALT levels in subgroup analysis (Table 4). However, we could not evaluate the exact normal ALT range where the negative ALT-mortality association was established. Fourth, the prevalence of death from liver-disease is extremely low (558 deaths, accounting for 3.43% of the overall occurrence) and the previous statistical data was mainly collected in younger adults. The close ALT-liver disease mortality association might have less clinical and epidemiological implications in the whole population, and the impact of age on the association between ALT level and liver disease-related mortality was difficult to evaluate. However, it provided a clue that an ALT assay might be sensitive and valuable in mortality prediction, especially in younger patients with liver disease. Fifth, mortality, not disease incidence was used as measured outcome. Mortality is not a direct presentation of the natural history of certain diseases and interfered by the socioeconomic, rural-urban, and health behavior disparities between different districts [67]-[69]. Clinicians should consider these covariates and draw discreet conclusions when faced with a given population. Notably, pooled HRs could not be evaluated by individual participant data, which is more reliable, with potential advantages [70]–[72]. Information (e.g., age, gender, follow-up duration, and HR) was extracted as aggregate data rather than individual participant data in respective study, which might cause larger heterogeneity and inconsistent analyses across studies [73]. Further meta-analysis using individual participant data is therefore needed. Despite these deficiencies, ALT is still a convenient and inexpensive assay for estimating the risk of death especially in older populations.

In conclusion, this systematic review and meta-analysis on prospective cohort studies provides evidence that ALT activity is strongly associated with liver disease-related mortality independent of viral infection in younger populations. Unlike the inconsistency of the ALT-mortality association in the younger subgroup, extremely low ALT levels indicated a high all-cause/disease-specific (including CV disease and cancer) mortality, especially within the normal ALT range in the older population. The ALT-mortality associations are influenced by age and the HRs of all-cause/disease-specific (CV disease and cancer) mortality caused by ALT elevation decreased by age. More well-designed studies with strict enrollment are needed to further confirm our findings.

## Supporting Information

Figure S1 **Flow chart of selected studies for meta-analysis.** Abbreviations: ALP: alkaline phosphatase; ALT: alanine aminotransferase; AST: aspartate aminotransferase; CVD: cardiovascular disease; DM: diabetes mellitus; GGT: gamma-glutamyl transpeptidase;ISI: Institute for Scientific Information; RR: relative risk.
(TIF)

Table S1 Search strategy for systematic review and meta-analysis of published literature on the association between alanine aminotransferase and all-cause/disease specific mortality in general population.
(DOC)

Table S2 Quality assessment of the studies included in the meta-analysis by NOS^a^. ^a^ “NOS” represented the Newcastle-Ottawa Scale. “1” meant the study was corresponded to the NOS criteria, “0” meant the study wasn't correspond to the NOS criteria.
(DOC)

Checklist S1 **PRISMA checklist.**
(DOC)

Flow Diagram S1 **PRISMA 2009 Flow Diagram.**
(DOC)
