# Supplementary figures and images for: Complex Association between Alanine Aminotransferase Activity and Mortality in General Population: A Systematic Review and Meta-Analysis of Prospective Studies
Source: PLoS One. 2014 Mar 14;9(3):e91410. doi: 10.1371/journal.pone.0091410 (PMC3954728; doi:10.1371/journal.pone.0091410)

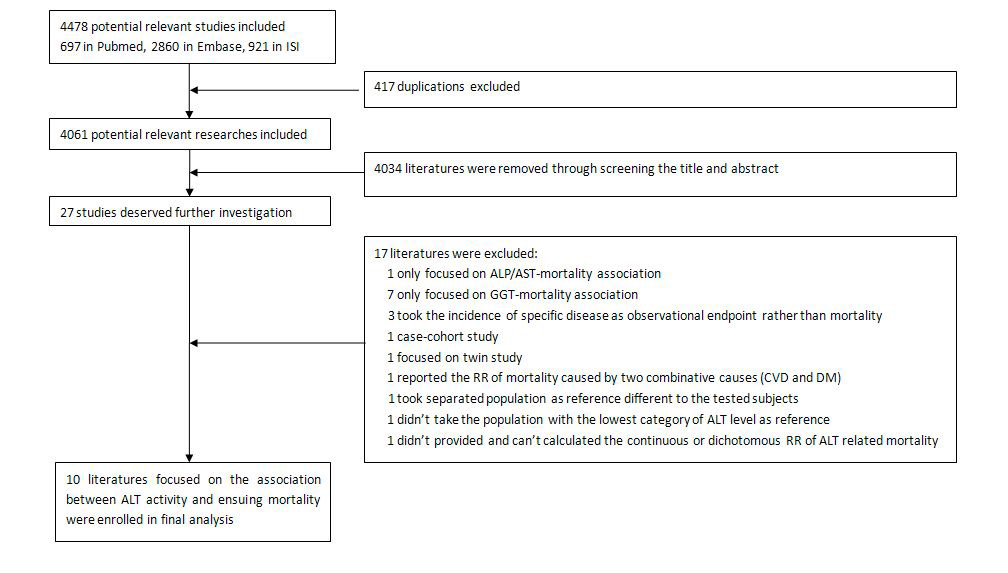

Supplement: Figure S1 — Flow chart of selected studies for meta-analysis. Abbreviations: ALP: alkaline phosphatase; ALT: alanine aminotransferase; AST: aspartate aminotransferase; CVD: cardiovascular disease; DM: diabetes mellitus; GGT: gamma-glutamyl transpeptidase;ISI: Institute for Scientific Information; RR: relative risk. (TIF) [file pone.0091410.s001.tif]
