# Supplementary material for: Complex Association between Alanine Aminotransferase Activity and Mortality in General Population: A Systematic Review and Meta-Analysis of Prospective Studies
Source: PLoS One. 2014 Mar 14;9(3):e91410. doi: 10.1371/journal.pone.0091410 (PMC3954728; doi:10.1371/journal.pone.0091410)
Supplement: Table S1 — Search strategy for systematic review and meta-analysis of published literature on the association between alanine aminotransferase and all-cause/disease specific mortality in general population. (DOC) [file pone.0091410.s002.doc]

Table S1: Search strategy for systematic review and meta-analysis of published literature on the association between alanine aminotransferase and all-cause/disease specific mortality in general population

1. Database: Pubmed

alanine aminotransferase [All Fields] OR alanine aminotransferase [MeSH] OR transaminase [MESH] OR transaminase [All Fields] AND mortality [MeSH]

#1. alanine aminotransferase

details: “alanine aminotransferase”[MeSH Terms] OR ("alanine"[All Fields] AND "aminotransferase"[All Fields]) OR "alanine aminotransferase"[All Fields]

#2. “transaminase” [MeSH Terms] OR “transaminase” [All Fields]

#3. #1 OR #2

#4. “mortality” [MeSH Terms]

#5. #3 AND #4

details：“alanine aminotransferase”[MeSH Terms] OR ("alanine"[All Fields] AND "aminotransferase"[All Fields]) OR "alanine aminotransferase"[All Fields] OR “transaminase” [MeSH Terms] OR “transaminase” [All Fields] AND “mortality” [MeSH Terms]

2. Database: Embase

#1. alanine aminotransferase

details：'alanine aminotransferase'/exp

#2. alanine transaminase

details: 'alanine transaminase '/exp

#3 glutamate pyruvate transaminase

details: ' glutamate pyruvate transaminase '/exp

#4. #1 OR #2 OR #3

#5. mortality

details: ' mortality '/exp

#6. [humans]/lim

#7. #4 AND #5 AND #6

details: 'alanine aminotransferase'/exp OR 'alanine transaminase'/exp OR 'glutamate pyruvate transaminase'/exp AND 'mortality'/exp AND [humans]/lim

3. Database: ISI all database

TI=("alanine aminotransferase" OR "alanine trensminase" OR "glutamate pyruvate transaminase" OR "alanine aminotransferase*" OR "alanine trensminase*" OR "glutamate pyruvate transaminase*") AND TI=("mortality" OR "mortality*") OR TS=("alanine aminotransferase" OR "alanine trensminase" OR "glutamate pyruvate transaminase" OR "alanine aminotransferase*" OR "alanine trensminase*" OR "glutamate pyruvate transaminase*") AND TS=("mortality" OR "mortality*")
