# Supplementary material for: Complex Association between Alanine Aminotransferase Activity and Mortality in General Population: A Systematic Review and Meta-Analysis of Prospective Studies
Source: PLoS One. 2014 Mar 14;9(3):e91410. doi: 10.1371/journal.pone.0091410 (PMC3954728; doi:10.1371/journal.pone.0091410)
Supplement: Table S2 — Quality assessment of the studies included in the meta-analysis by NOSa. a “NOS” represented the Newcastle-Ottawa Scale. “1” meant the study was corresponded to the NOS criteria, “0” meant the study wasn't correspond to the NOS criteria. (DOC) [file pone.0091410.s003.doc]

Table S2 Quality assessment of the studies included in the meta-analysis by NOS^a^

| NOS scale | Arndt  et al [8] (1998) | Kim et al [3] (2004) | Elinav  et al [15] (2006) | Nalamura  et al [4] (2006) | Schindhelm et al [9] (2007) | Ruhl et al [11] (2009) | Hovinen et al [17]  (2010) | Ford et al WOSCOPS [13] (2011) | Ford et al PROSPER [13](2011) | Ford et al Leiden 85-plus [13](2011) | Schooling et al [14] (2012) | Koehler et al [18] (2013) |
| --- | --- | --- | --- | --- | --- | --- | --- | --- | --- | --- | --- | --- |
| **A Selection (maximum 4)** |  |  |  |  |  |  |  |  |  |  |  |  |
| **1.Representativeness of general community population** | 0 | 1 | 0 | 1 | 1 | 1 | 0 | 0 | 0 | 0 | 1 | 1 |
| **2.The reference group was drawn from the same community** | 1 | 1 | 1 | 1 | 1 | 1 | 1 | 1 | 1 | 1 | 1 | 1 |
| **3.Ascertainment the exposure of high ALT activity** | 1 | 1 | 1 | 1 | 1 | 1 | 1 | 1 | 1 | 1 | 1 | 1 |
| **4.Death was not present at baseline** | 1 | 1 | 1 | 1 | 1 | 1 | 1 | 1 | 1 | 1 | 1 | 1 |
| **B Comparability (maximum 2)** |  |  |  |  |  |  |  |  |  |  |  |  |
| **5.Controlled for age and gender** | 1 | 1 | 1 | 1 | 1 | 1 | 0 | 1 | 1 | 1 | 1 | 1 |
| **6.Controlled for 2 or more variables including BMI, alcohol intake, and cigarette smoking** | 1 | 1 | 0 | 1 | 0 | 1 | 1 | 1 | 1 | 1 | 0 | 1 |
| **C Outcome (maximum 3)** |  |  |  |  |  |  |  |  |  |  |  |  |
| **7. Death was certificated by hospital or local municipal registration** | 1 | 1 | 1 | 1 | 1 | 1 | 1 | 1 | 1 | 1 | 1 | 1 |
| **8. Adequate duration of follow-up (≥5 years)** | 1 | 1 | 1 | 1 | 1 | 1 | 1 | 0 | 0 | 0 | 1 | 1 |
| **9. Adequacy of follow-up rate (>90%) of cohorts** | 1 | 1 | 1 | 1 | 0 | 0 | 0 | 1 | 1 | 1 | 1 | 1 |
| **Total scores (maximum 9)** | 8 | 9 | 7 | 9 | 7 | 8 | 6 | 7 | 7 | 7 | 8 | 9 |

^a^ “NOS” represented the Newcastle-Ottawa Scale

“1” meant the study was corresponded to the NOS criteria,” 0” meant the study wasn’t correspond to the NOS criteria
