# Supplementary material for: Complex Association between Alanine Aminotransferase Activity and Mortality in General Population: A Systematic Review and Meta-Analysis of Prospective Studies
Source: PLoS One. 2014 Mar 14;9(3):e91410. doi: 10.1371/journal.pone.0091410 (PMC3954728; doi:10.1371/journal.pone.0091410)
Supplement: Flow Diagram S1 — PRISMA 2009 Flow Diagram. (DOC) [file pone.0091410.s005.doc]

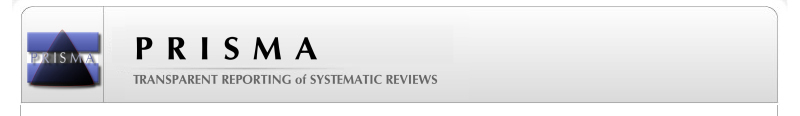
**PRISMA 2009 Flow Diagram**

**Screening**

**Included**

**Eligibility**

**Identification**

Records identified through database searching
(n = 4478 )

Additional records identified through other sources
(n = 0 )

Records after duplicates removed
(n = 417)

Records screened
(n = 4061)

Records excluded
(n = 4034 )

Full-text articles assessed for eligibility
(n = 27)

Full-text articles excluded, with reasons
(n = 17 )

Literatures included in qualitative synthesis
(n = 10 )

Literatures included in quantitative synthesis (meta-analysis)
(n = 10 )
